# APOL1 genotyping via buccal mucosa cell samples to establish risk of kidney disease

**DOI:** 10.1186/s12882-022-02954-w

**Published:** 2022-10-10

**Authors:** Dona Jeanne Alladagbin, Carlos Gustavo Regis da Silva, Luciano Kalabric Silva, Washington LC dos-Santos, Geraldo Gileno de Sá Oliveira

**Affiliations:** Fundação Oswaldo Cruz, Laboratory of Molecular and Strutural Pathology (LAPEM), Gonçalo Moniz Institute, FIOCRUZ-BA, Rua Waldemar Falcão, no. 121, CEP 40.296-710 Salvador, Brazil

**Keywords:** Buccal mucosa cells, Blood, PCR, DNA sequencing, Apolipoprotein L, Haplotypes

## Abstract

Two alleles (G1 and G2) of the apolipoprotein 1 gene (*APOL1*) predispose people of African descent to developing or accelerating the course of certain types of kidney disease. Population studies to determine the frequency of the G1 and G2 alleles are important to inform resource allocation by public health authorities. Traditionally, *APOL1* genotyping is carried out in blood samples. However, sample collection, transport, and storage is cumbersome. Here we compared *APOL1* genotyping in blood and buccal mucosa cell samples obtained from 23 individuals. Alleles G0 (wild), G1, and G2, as well as genotypes G0/G0, G0/G1, G1/G1, G0/G2, G1/G2, and G2/G2 were detected using both blood and buccal mucosa cells with 100% coincidence. Our data indicate that buccal mucosa cell samples may represent a suitable alternative to blood samples for *APOL1* genotyping in the field.

**Supplementary Information:**

The online version contains supplementary material available at 10.1186/s12882-022-02954-w.

## Introduction

Apolipoprotein 1 (*APOL1*) is a component of both high-density lipoprotein (HDL) [1] and the innate immune system; with regard to the latter, it functions as a protective factor against human African trypanosomiasis (HAT), which causes African sleeping sickness [2, 3]. The *APOL1* gene is located on chromosome 22 and encodes a protein 398 amino acids in length [4]. Three alleles of *APOL1* have been described, G0 or wild-type, G1, and G2. Alleles G1 and G2 emerged as a point mutation and a deletion in the *APOL1* gene segment encoding the domain involved in interaction with the trypanosome serum resistance-associated protein (SRA), respectively [3, 5]. Homozygosity for either G1 or G2, as well as compound heterozygosity (G1/G2), predispose individuals of African descent to the development or accelerated progression of renal diseases associated with hypertension, focal segmental glomerulosclerosis (FSGS), as well as nephropathies associated with viral infections, such as HIV and SARS-CoV-2 [3, 6]. Furthermore *APOL-1* risk variants also associate with decrease in kidney allograft survival. Given the growing importance of *APOL1* polymorphism in defining the prognosis of kidney diseases, estimating the frequency of G1 and G2 alleles in populations of African descent is of paramount concern. In clinical medicine, the detection of these biomarkers can aid prognostic determination and guide therapeutic strategies in renal diseases. In addition, informing the detection of *APOL1* variants, which are known to influence kidney disease risk, can help direct health resources and implement policies that promote well-being in populations of African descent. The current method used to identify *APOL1* risk alleles involves gene segment amplification by PCR and DNA sequencing in blood samples [7] or tissue fragments. However, the invasive sample collection approach results in low participation rates in research studies. Moreover, these samples also require special handling, transport, and storage conditions. Herein, we describe a preliminary study on the use of buccal mucosa cells, collected via flocked swabs, for DNA sequencing to determine the presence of *APOL1* allele variants.

## Materials and methods

### Patients

This study was carried out in a convenience sample of 23 patients who underwent renal biopsy to diagnose nephropathy at the Nephrology Service of the Ana Nery Hospital (HAN) in Salvador, Brazil. Patients were of both sexes, with age ranging between 15 and 62 years.

### Ethics statement

This study was carried out in accordance with recommendations established by the Brazilian National Health Council (466/2012) and received approval from the Institutional Review Board for Research involving Human Subjects, Gonçalo Moniz Institute (Fiocruz-BA, protocol number 382.273). Blood samples and buccal mucosa cells were collected following the provision of written consent from either patients or their legal guardians.

### Collection, storage, and transport of blood and buccal mucosa cell samples

Blood samples were collected by venipuncture, transported on ice, and stored at -4 ^o^C until the time of DNA extraction. Buccal mucosa cell samples were collected and transported in accordance with a previously described protocol [8].

### DNA extraction, PCR amplification, and electrophoresis

Blood (5 mL) was collected from each patient in EDTA tubes. A sample of buccal mucosal cells was also obtained from each patient by scraping the cheek mucosa with a flocked swab. After scraping, the shaft was broken and the flocked tip was placed in a sterile 1.5 mL Eppendorf tube. These tubes were placed on ice in a Styrofoam container and transported for laboratory analysis. Oral mucosal cells were eluted from each swab using 400 µL of phosphate-buffered saline (PBS). Either 200 µL of blood or 400 µL of oral mucosal cell suspension were submitted to DNA extraction using a QIAamp DNA Mini kit or a DNeasy Blood and Tissue kit (Qiagen, Valencia, USA), respectively, in accordance with the manufacturer’s instructions. After purification, DNA samples were qualitatively and quantitatively analyzed on a nanodrop apparatus (Thermo Fisher Scientific, Wilmington, USA). Polymerase chain reactions were performed using extracted DNA and high-fidelity DNA polymerase, as previously described (8). The amplified PCR products were detected on 1% agarose gel containing 1 µg/mL ethidium bromide.

### ***APOL1*genotyping**

Amplified PCR products were treated with ExoSAP-IT Cleanup Reagent and sequenced using the Sanger method as previously described [7, 9]. The forward and reverse DNA sequences were aligned using Genbank reference BC143038 and CLC Main Workbench v.8.0 software (Qiagen). Conflicting sites were confirmed by visual inspection of electropherograms. Three markers were analyzed: rs73885319, rs60910145, and rs71785313. Diallelic SNPs rs73885319 [A/G] and rs60910145 [T/G] were genotyped as homozygous in the presence of a single peak, and heterozygous when a double peak was identified in both sequences. Rs71785313 insdel [-/ATAATT/TTATAA] was genotyped as homozygous or heterozygous, respectively, when sequences aligned perfectly in both directions, or aligned up to the deletion site, and thereafter showed overlapping nucleotides. Genotyping results for each patient sample, blood or buccal mucosa cells, were recorded on an Excel spreadsheet, with risk allele variants deduced whenever possible as G1^GM^ [G-G-Ins], G1^GI^ [G-T-Ins], G2 [A-T-Del], or wild-type G0 [A-T-Ins]; relative frequencies were subsequently calculated [9].

### Statistical analysis

Data are reported as absolute values or percentages and summarized as medians with 1st and 3rd quartiles. Agreement between the genotype results from blood or buccal mucosa cell samples was evaluated by Cohen’s kappa test [10]. Kappa values below 0, 0-0.2, 0.21–0.4, 0.41–0.6, 0.61–0.8 and 0.81-1 were respectively considered as: no agreement, slight agreement, fair agreement, moderate agreement, substantial agreement, and almost perfect agreement.

## Results

### Study population

The main demographic and clinical characteristics of the studied population are shown in Table 1. Patients ranged in age from 15 to 62 years, and 19 (83%) were female. Most were diagnosed with lupus nephritis or FSGS.

**Table 1:** Clinical and demographic data on patients undergoing renal biopsy for the diagnosis of glomerular disease in Salvador, Brazil

Parameter	Value	(%) [1st-3rd quartiles]
Total number	23	(100%)
Age in years (median)	25	[20–37]
Age range	15–62	
Female	19	(83%)
Self-reported skin color		
Black	12	(52%)
Mixed-race	10	(44%)
White	1	(4%)
Clinical presentation		
Systemic Arterial Hypertension	20	(87%)
Nephrotic range proteinuria	14	(64%)
Non-nephrotic proteinuria	8	(36%)
Renal failure	8	(35%)
Diabetes mellitus	3	(13%)
Laboratory results (median)		
Albumin (g/dL)	2.6	[1.7–3.4]
Creatinine (mg/dL)	0.9	[0.7–1.6]
Urea (mg/dL)	41	[32–55]
Total cholesterol (mg/dL)	236	[198–374]
24-hour proteinuria (g/24 h)	4.7	[1.5–12.4]
Histological diagnosis:		
Lupus nephritis	9	(39%)
Focal and segmental glomerular sclerosis	5	(22%)
Membranous glomerulopathy	3	(13%)
IgA Nephropathy	2	(9%)
Membranoproliferative glomerulonephritis	1	(4%)
Focal glomerulonephritis	1	(4%)
Vasculitis of small arteries	1	(4%)
Insufficient sample material for diagnosis	1	(4%)

### **PCR amplification of*APOL1*in buccal mucosa cell samples**

**Fig. 1:**
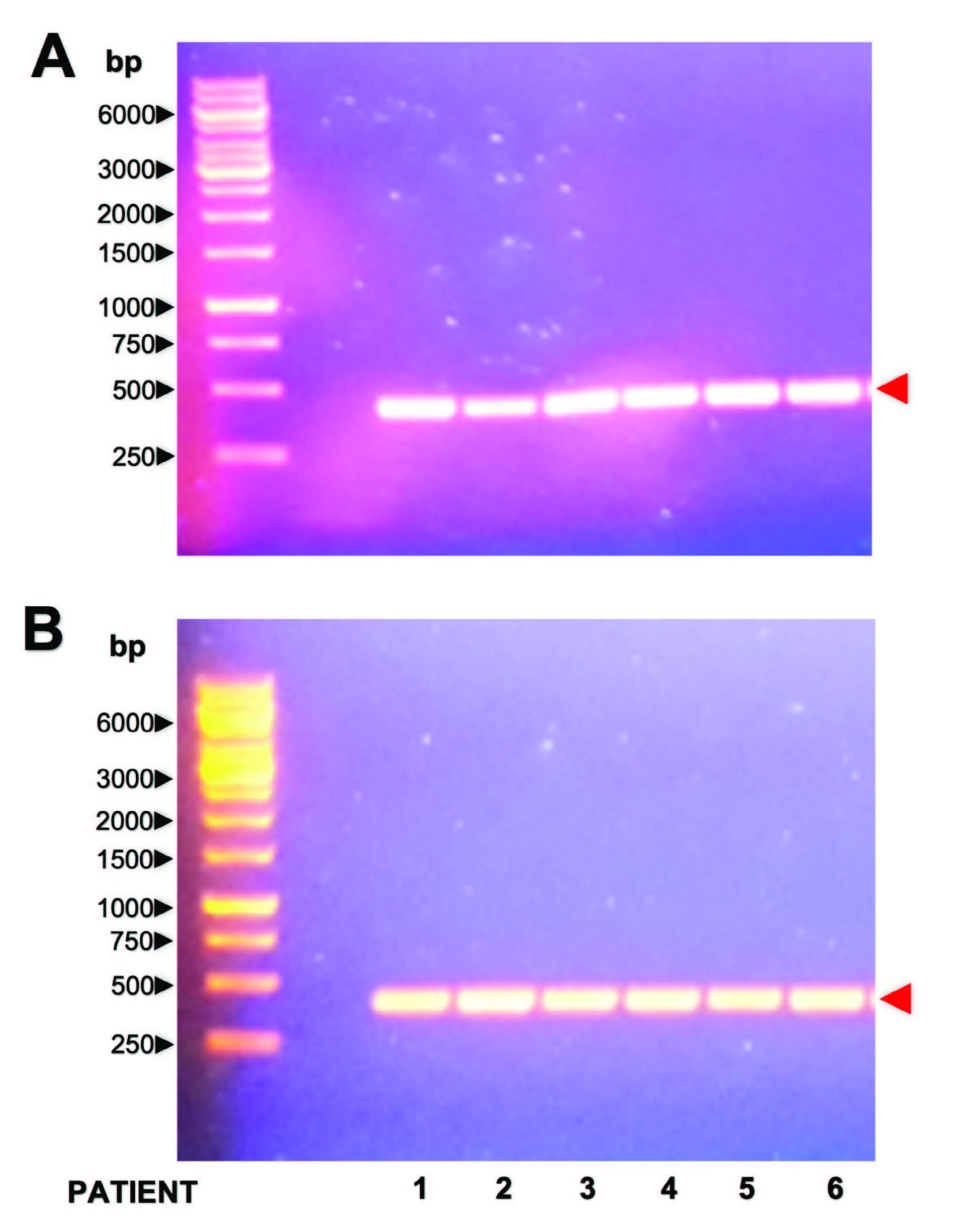
Analysis of PCR products obtained from the amplification of an *APOL1* gene segment by agarose gel electrophoresis PCR was carried out using DNA extracted from blood and buccal mucosa cell samples from all 23 patients. PCR products (10µL) from blood (A) and buccal mucosa cell samples (B) were analyzed by agarose gel electrophoresis using 1% agarose gels containing 1 µg/mL ethidium bromide. Representative examples of the tested samples are shown. Black arrowheads indicate molecular weight markers, while red arrowheads correspond to amplified PCR products (DNA bands approximately 422 bp long), respectively

Blood samples have previously been successfully used for *APOL1* genotyping [9]. To determine whether mucosal cell samples could also be used for this purpose, PCRs were performed to amplify a 422 bp DNA segment of *APOL1* in blood samples (positive control) and buccal mucosal cells samples obtained from 23 patients. This DNA segment encodes part of the membrane-addressing domain and the whole SRA-domain of the APOL1 protein. PCR products analyzed by agarose gel electrophoresis revealed DNA bands approximately 422 bp in length (Fig. 1).

### **Characterization of*APOL1*SNPs, insdel, and alleles in buccal cell samples**

To confirm that *APOL1* genotyping could be performed using buccal mucosa cell samples, PCR products were treated with ExoSAP-IT and sequenced in both directions by the Sanger method. The DNA sequences obtained were analyzed using Genbank reference BC143038 and CLC Main Workbench v.8.0 software (Qiagen). SNPs/insdel rs73885319, rs60910145, and rs71785313 were identified (Table 2) as previously described [7, 9]. Using the identified SNPs/insdel, the alleles (G0, G1, and G2) of *APOL1* were defined for each patient’s blood and buccal mucosa cell samples (Table 2). A comparison of the resulting chromatograms revealed 100% coincidence in the SNPs/insdel findings (Supplemental figure) for all pairs of blood and buccal mucosa cell samples from the 23 patients. Six different genotypes were identified among the patients (Table 2), with most (n = 16) classified as G0/G0, three as G0/G2 and one each of G0/G1, G1/G1, G1/G2, and G2/G2. Correlation analysis between the genotypes ascribed to blood and buccal mucosa cell samples, as evaluated by Cohen’s kappa test, revealed a coefficient equal to 1, indicating almost perfect agreement.

**Table 2:** Characterization of SNPs/indel and haplotypes of *APOL1* in blood and buccal mucosa cell samples from 23 patients

Id #	SNPs/Insdel						Haplotypes	
	rs73885319 [A/G]		rs60910145 [G/T]		rs71785313 [-/ATAATT/TTATAA]		Whole blood	Buccal mucosa cells
Patient 1	1	1	4	4	2	2	G0/G0	G0/G0
Patient 2	1	3	3	4	2	2	G0/G1	G0/G1
Patient 3	3	3	3	3	2	2	G1/G1	G1/G1
Patient 4	1	1	4	4	1	2	G0/G2	G0/G2
Patient 5	1	1	4	4	1	1	G2/G2	G2/G2
Patient 6	1	3	3	4	1	2	G1/G2	G1/G2
Patient 7	1	1	4	4	2	2	G0/G0	G0/G0
Patient 8	1	1	4	4	2	2	G0/G0	G0/G0
Patient 9	1	1	4	4	2	2	G0/G0	G0/G0
Patient 10	1	1	4	4	1	2	G0/G2	G0/G2
Patient 11	1	1	4	4	1	2	G0/G2	G0/G2
Patient 12	1	1	4	4	2	2	G0/G0	G0/G0
Patient 13	1	1	4	4	2	2	G0/G0	G0/G0
Patient 14	1	1	4	4	2	2	G0/G0	G0/G0
Patient 15	1	1	4	4	2	2	G0/G0	G0/G0
Patient 16	1	1	4	4	2	2	G0/G0	G0/G0
Patient 17	1	1	4	4	2	2	G0/G0	G0/G0
Patient 18	1	1	4	4	2	2	G0/G0	G0/G0
Patient 19	1	1	4	4	2	2	G0/G0	G0/G0
Patient 20	1	1	4	4	2	2	G0/G0	G0/G0
Patient 21	1	1	4	4	2	2	G0/G0	G0/G0
Patient 22	1	1	4	4	2	2	G0/G0	G0/G0
Patient 23	1	1	4	4	2	2	G0/G0	G0/G0

A = 1, C = 2, G = 3; T = 4, Del = 1, ATAATT = 2, and TTATAA = 3. G0 = A-T-Ins; G1^GM^ = G-G-Ins, G1^GI^ = G-T-Ins, and G2 = A-T-Del.

## Discussion

The present report evaluated the feasibility of using buccal mucosa cells as a proxy for blood samples to perform *APOL-1* genotyping evaluated.

Samples of variable origin (secretions, tissues, organs, etc.) and various methods (including PCR, restriction fragment length polymorphism-RFLP, random amplified polymorphic detection-RAPD, amplified fragment length polymorphism-AFLP, DNA sequencing, allele-specific oligonucleotide (ASO) probes and microarray analysis) can be used for genotyping, allowing for the identification of genetic variations among individuals [11, 12]. Each sample type and method presents unique advantages and disadvantages. Traditionally, blood samples are most commonly used for genetic studies. In these samples, the DNA extracted from white blood cells is generally of high quality, leading to elevated genotyping success rates. However, blood is also cumbersome to handle. As a more feasible alternative, mucosa cells were evaluated herein with respect to *APOL1* genotyping potential. Compared to blood collection and handling, buccal mucosa cell sampling presents several advantages, e.g., the use of a flocked swab is rapid, minimally-invasive, and painless; moreover, the transportation and storage of mucosa cell samples is simpler and less expensive than handling blood samples.

Paired blood (positive control) and buccal mucosa cell samples from 23 patients submitted to biopsy for the diagnosis of kidney disease were used for genotyping. The latter are composed of epithelial cells and leukocytes, but may also contain oral flora [13]. In addition, swabbing may also capture dead buccal mucosa cells [13]. The presence of bacteria and dead cells could contribute to the partial DNA degradation seen in this type of sample, thus leading to PCR amplification failure [14]. Indeed, assays relying on the amplification of long DNA segments (~ 10 kbp) by PCR, such as HLA genotyping, in buccal mucosa cell samples can fail due to partial DNA degradation (14). By contrast, the amplification of relatively short DNA fragments by PCR in buccal mucosa samples has been reported to be reliable [15].

Our results demonstrate the successful amplification of a DNA segment in the *APOL1* gene in 23 buccal mucosa cell samples collected via flocked swab (Fig. 1), suggesting that the DNA obtained from this sampling technique is suitable for *APOL1* genotyping. In addition, the analysis of chromatograms and nucleotide sequences following Sanger sequencing revealed all possible *APOL1* alleles (G0, G1, and G2) and genotypes (G0/G0, G0/G1, G0/G2, G1/G1, and G1/G2, and G2/G2) in the 23 buccal mucosa cell samples evaluated (Supplementary Figure and Table 2). Correlation analysis between genotypes obtained from blood and buccal mucosa cell samples demonstrated almost perfect agreement (Cohen´s correlation coefficient = 1.0).

Taken together, the data presented herein indicate that the quality of DNA obtained from buccal mucosa cells collected via flocked swabs is of sufficient quality to reliably perform *APOL1* genotyping. We therefore suggest that buccal mucosa cells samples represent a suitable alternative to blood samples for *APOL1* genotyping purposes.

One limitation of this study is related to the small sample size used to carry out the genotyping. Although there was complete agreement between genotypes of *APOL1* in paired samples from buccal mucosa cells and blood in 23 individuals, it is worth analyzing a larger number of samples from individuals in different clinical settings to confirm the reported results.

Finally, we hope that the technical approach presented herein may contribute to integrate *APOL-1* screening to clinical nephrology practice.

## Conclusion

Buccal mucosa cell samples obtained via flocked swab may be used in place of blood samples for *APOL1* genotyping, thus facilitating the performance of population studies.

## Electronic supplementary material

Below is the link to the electronic supplementary material.

Supplementary Material 1

## Data Availability

All relevant data and materials were described in the manuscript and supplementary figure. Any addition information can be requested directly to the corresponding author. DNA sequences and the corresponding translation sequences are deposited under Genbank accession numbers ON325439 to ON325484. The web links of gene bank are: https://www.ncbi.nlm.nih.gov/nuccore/ON325439. https://www.ncbi.nlm.nih.gov/nuccore/ON325440. https://www.ncbi.nlm.nih.gov/nuccore/ON325441. https://www.ncbi.nlm.nih.gov/nuccore/ON325442. https://www.ncbi.nlm.nih.gov/nuccore/ON325443. https://www.ncbi.nlm.nih.gov/nuccore/ON325444. https://www.ncbi.nlm.nih.gov/nuccore/ON325445. https://www.ncbi.nlm.nih.gov/nuccore/ON325446. https://www.ncbi.nlm.nih.gov/nuccore/ON325447. https://www.ncbi.nlm.nih.gov/nuccore/ON325448. https://www.ncbi.nlm.nih.gov/nuccore/ON325449. https://www.ncbi.nlm.nih.gov/nuccore/ON325450. https://www.ncbi.nlm.nih.gov/nuccore/ON325451. https://www.ncbi.nlm.nih.gov/nuccore/ON325452. https://www.ncbi.nlm.nih.gov/nuccore/ON325453. https://www.ncbi.nlm.nih.gov/nuccore/ON325454. https://www.ncbi.nlm.nih.gov/nuccore/ON325455. https://www.ncbi.nlm.nih.gov/nuccore/ON325456. https://www.ncbi.nlm.nih.gov/nuccore/ON325457. https://www.ncbi.nlm.nih.gov/nuccore/ON325458. https://www.ncbi.nlm.nih.gov/nuccore/ON325459. https://www.ncbi.nlm.nih.gov/nuccore/ON325460. https://www.ncbi.nlm.nih.gov/nuccore/ON325461. https://www.ncbi.nlm.nih.gov/nuccore/ON325462. https://www.ncbi.nlm.nih.gov/nuccore/ON325463. https://www.ncbi.nlm.nih.gov/nuccore/ON325464. https://www.ncbi.nlm.nih.gov/nuccore/ON325465. https://www.ncbi.nlm.nih.gov/nuccore/ON325466. https://www.ncbi.nlm.nih.gov/nuccore/ON325467. https://www.ncbi.nlm.nih.gov/nuccore/ON325468. https://www.ncbi.nlm.nih.gov/nuccore/ON325469. https://www.ncbi.nlm.nih.gov/nuccore/ON325470. https://www.ncbi.nlm.nih.gov/nuccore/ON325471. https://www.ncbi.nlm.nih.gov/nuccore/ON325472. https://www.ncbi.nlm.nih.gov/nuccore/ON325473. https://www.ncbi.nlm.nih.gov/nuccore/ON325474. https://www.ncbi.nlm.nih.gov/nuccore/ON325475. https://www.ncbi.nlm.nih.gov/nuccore/ON325476. https://www.ncbi.nlm.nih.gov/nuccore/ON325477. https://www.ncbi.nlm.nih.gov/nuccore/ON325478. https://www.ncbi.nlm.nih.gov/nuccore/ON325479. https://www.ncbi.nlm.nih.gov/nuccore/ON325480. https://www.ncbi.nlm.nih.gov/nuccore/ON325481. https://www.ncbi.nlm.nih.gov/nuccore/ON325482. https://www.ncbi.nlm.nih.gov/nuccore/ON325483. https://www.ncbi.nlm.nih.gov/nuccore/ON325484.
