# Supplementary material for: APOL1 genotyping via buccal mucosa cell samples to establish risk of kidney disease
Source: BMC Nephrol. 2022 Oct 10;23:329. doi: 10.1186/s12882-022-02954-w (PMC9549854; doi:10.1186/s12882-022-02954-w)
Supplement: Supplementary file 1 — Supplementary Material 1 [file 12882_2022_2954_MOESM1_ESM.docx]

**Supplemental figure**: Comparison of DNA sequences obtained from blood samples and buccal mucosa cells used for *APOL1* genotyping. Electropherograms illustrate different *APOL1* genotypes in pairs of blood and buccal mucosa cell samples. Sanger sequencing can differentiate homozygous and heterozygous SNPs by the presence of a single peak or double peaks, respectively, in forward or reverse sequences. It can also differentiate homozygous insdel when the insertion region is sequenced, or not, in both forward and reverse directions. By contrast, heterozygous insdel can only be identified in forward sequences due to noise created by overlap in the ins or del allele sequences. Usually, the region beyond the insdel is trimmed by most software applications due to poor quality. Since the reverse primer is usually next to the insdel, almost the entire reverse sequence becomes noisy, thus preventing alignment using the forward sequence.

Lower case letters = conflict resolution or ambiguity in nucleotide identification; Red = reverse sequences; Green = forward sequences.

*For some blood and buccal mucosa cells samples, only forward sequences were available for analysis. Dashed lines indicate the positions of polymorphisms.
